# Harmonic FMCW Radar System: Passive Tag Detection and Precise Ranging Estimation

**DOI:** 10.3390/s24082541

**Published:** 2024-04-15

**Authors:** Ahmed El-Awamry, Feng Zheng, Thomas Kaiser, Maher Khaliel

**Affiliations:** 1 Institute of Digital Signal Processing, University of Duisburg-Essen, 47057 Duisburg, Germany; 2 Benha Faculty of Engineering, Benha University, Benha 13512, Egypt

**Keywords:** harmonic FMCW radar, passive harmonic transponder, range estimation, power consumption, simulation, measurement

## Abstract

This paper details the design and implementation of a harmonic frequency-modulated continuous-wave (FMCW) radar system, specialized in detecting harmonic tags and achieving precise range estimation. Operating within the 2.4–2.5 GHz frequency range for the forward channel and 4.8–5.0 GHz for the backward channel, this study delves into the various challenges faced during the system’s realization. These challenges include selecting appropriate components, calibrating the system, processing signals, and integrating the system components. In addition, we introduce a single-layer passive harmonic tag, developed specifically for assessing the system, and provide an in-depth theoretical analysis and simulation results. Notably, the system is characterized by its low power consumption, making it particularly suitable for short-range applications. The system’s efficacy is further validated through experimental evaluations in a real-world indoor environment across multiple tag positions. Our measurements underscore the system’s robust ranging accuracy and its ability to mitigate self-interference, showcasing its significant potential for applications in harmonic tag detection and ranging.

## 1. Introduction

Radar systems play a pivotal role in various applications, such as target detection, ranging, and tracking. In recent years, frequency-modulated continuous-wave (FMCW) radar has gained significant attention due to its superior range resolution and accuracy. However, conventional FMCW radar encounters challenges from self-interference and clutter reflections, rendering the detection of small targets or tags quite challenging. Moreover, these undesired environmental clutter reflections are considered interference signals, diminishing the signal-to-interference ratio (SIR) and subsequently affecting ranging accuracy. This research exploits a radar principle known as harmonic FMCW radar, leveraging the harmonic properties of the transmitted waveform to enhance the radar system’s performance [1]. Notably, the target (harmonic tag) incorporates a nonlinear device that converts the incidence fundamental frequencies into desired harmonic frequencies. Consequently, the tag response can be uniquely differentiated from the predominantly linear environmental clutter reflections. Therefore, the harmonic radar is only detecting targets that exhibit non-linear behavior, specifically when the backscattering signal contains second harmonic frequencies. Such a radar cannot assist in detecting simple metallic reflectors, and thus the clutter signal is mitigated.

While harmonic FMCW radar presents numerous advantages, it also poses challenges in beat frequency measurement. The following challenges are associated with harmonic FMCW beat frequency measurement:

1. Nonlinearities of the transmitted signal: In practical scenarios, imperfections of radar hardware can introduce harmonics (multiples of the fundamental frequency) into the received signal. While harmonic generation is the principle used in harmonic radar, excessive or unintended harmonics can interfere with backscattered harmonic signals from the tag. Thus, the self-interference will be much stronger than the harmonic signal generated from the tag, which will deteriorate the received signal.
2. Frequency drift: FMCW radar accuracy relies on precise frequency measurements. However, factors like temperature variations and oscillator stability can introduce frequency drift. Such drift can lead to errors in beat frequency measurement, consequently yielding inaccurate distance estimation.
3. Multipath interference: FMCW radar signals are susceptible to multipath interference, wherein the transmitted signal reflects off multiple surfaces before reaching the receiver. These reflected signals can interfere with the direct signal, distorting the received signal and affecting the accuracy of the beat frequency measurements by introducing errors in distance calculations.
4. Signal-to-noise ratio (SNR): The accuracy of FMCW beat frequency measurement is influenced by the SNR of the received signal. In scenarios with low SNR, detecting the beat frequency becomes challenging, resulting in increased measurement errors. Environmental factors, such as clutter, interference, and attenuation, can degrade the SNR and compromise the reliability of the beat frequency measurement. In harmonic radar systems, the nonlinear element at the tag’s side provides a significant conversion loss (which will be discussed later in the system model section), which provides an extra loss on top of the path loss. This makes the SNR parameter more challenging in the passive harmonic radar system.

To counter these challenges, various techniques are employed, including signal processing algorithms, calibration procedures, and advanced hardware design, as in [2,3,4]. These techniques aim to compensate for nonlinearities, reduce frequency drift, mitigate multipath interference, and enhance overall SNR. Continuous research and advancements in radar technology persist in addressing these challenges, subsequently enhancing the accuracy and robustness of FMCW beat frequency measurements. Typical radio frequency communication systems suffer from similar challenges, but it also provides more flexible solutions to overcome these challenges, such as coding gain, error detection, and corrections techniques that cannot be used in radar systems.

**Figure 1:**
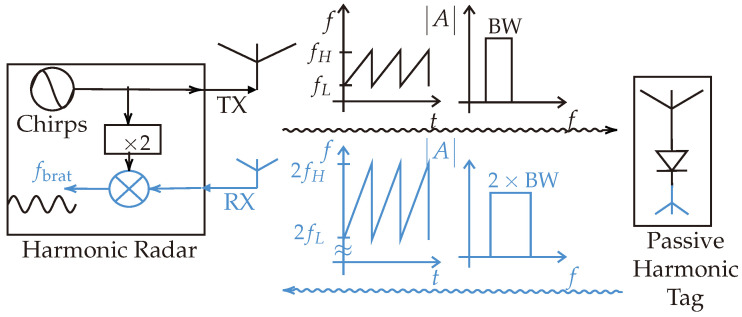
Principal block diagram. The forward path is marked in black, and the backward path is marked in blue. The backscattered signal from the passive harmonic tag has twice the bandwidth of the transmitted signal.

Figure 1 shows the principal block diagram of the harmonic FMCW radar. The backscattered signal from the passive harmonic tag has a total bandwidth twice that of the transmitted signal. Consequently, range accuracy will be improved.

**Figure 2:**
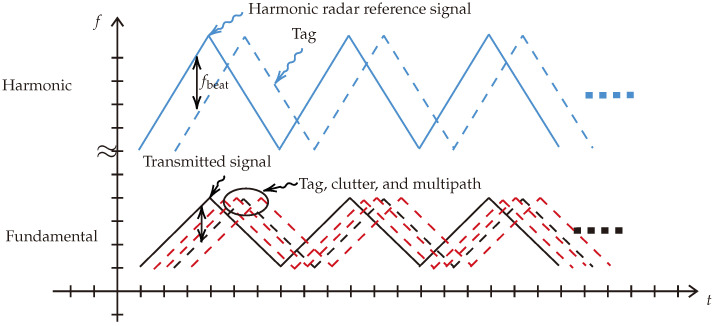
Proposed system functionality illustration. The dashed black and red curves represent the reflected signals in the fundamental frequency domain that will be received by a linear radar. The blue and dashed blue curves represent the backscattered chirps in the harmonic domain that will be received by the harmonic radar.

In principle, the system transmits continuous frequency ramps, as illustrated in Figure 2, which also shows the backscattered clutter signals in the fundamental channel. The transmitted ramp’s slope is B/T, where *B* represents the signal bandwidth, and *T* signifies the ramp sweeping time. The backscattered signal from the harmonic tag is located in the harmonic channel of ramp slope 2B/T, representing a clutter-free signal.

Prior research has considered the realization of harmonic radar to estimate the range of harmonic objects. The system introduced in [1] describes the design and implementation of a harmonic radar system (from 60 to 64 GHz) using self-designed monolithic microwave integrated circuits (MMICs) for an active harmonic tag. The system proposed in [5] relies on an implemented MMIC (from 78 to 80 GHz) and a realized active harmonic tag to detect pedal cyclists in outdoor scenarios. The utilization of millimeter wave optical harmonic transponders for tracking small insects is discussed in [6]. It mentions the prototype radar operating in the CW mode of frequency 38.5 GHz and its detection range of up to 1 m with an active tag. These research studies use active harmonic tag designs to compensate for the high loses at the utilized frequencies. Moreover, there are some other harmonic radar realizations with passive tags. In [7], two compact low-cost FMCW harmonic radar prototypes for insect tracking are proposed. They are operating at the S-band and the X-band with output power of 3 W and 10 W, respectively. In addition, a portable low-power harmonic radar system is introduced in [8]. The radar unit transmits a 5.9–6 GHz signal and detects the 11.8–12 GHz harmonic created by the passive tag. Most of these research studies consider an outdoor scenario, whereas our proposed system focuses on an indoor test case. Furthermore, aside from the previous work, our proposed system consider the well-known industrial, scientific, and medical (ISM) band of 2.4 to 2.5 GHz. The proposed harmonic radar realization in this paper differs from the aforementioned research in that (i) the entire design is implemented on one PCB with off-the-shelf components, (ii) the system design provides a high mitigation of the generated harmonics from the transmitter, and (iii) the introduced system design provides a low phase noise, which negatively affects the performance of FMCW systems.

In this article, we introduce a practical realization of harmonic FMCW system components, including the reader (radar system) and the passive harmonic tag (target). The aforementioned challenges are meticulously considered in the hardware design, resulting in a system suitable for real-world applications and environments. Furthermore, the tag is designed to be easily affixed to actual products. Additionally, the measurements are conducted in an indoor environment with the presence of clutter signals.

This paper is structured as follows: Section 2 lists several harmonic radar applications. Section 3 illustrates the design of the passive harmonic tag. Section 4 presents the system model. Section 5 introduces the proposed harmonic radar realization. The simulation results of the proposed system model are discussed in Section 6, and the real measurements are presented in Section 7. Finally, conclusions are drawn in Section 8.

## 2. Applications of Harmonic Radar

A review of recent achievements in harmonic radar development is provided in [9,10,11]. As previously mentioned, harmonic radar is a radar system type that leverages targets’ nonlinear properties to enhance detectability. This system finds applications in various fields due to its distinct characteristics. Notably, one common application is tracking insects, particularly in ecology and entomology. Insects are affixed with small transponders reflecting a harmonic of the incident frequency. This enables researchers to track individual insects over substantial distances, yielding valuable behavior data like migration and foraging [7,12,13,14,15].

Harmonic radar is also instrumental in locating lost or stranded individuals in environments with poor visibility, such as heavy snow or wooded areas. Individuals carry small transponders triggered by radar signals, emitting harmonics of the incident frequency detectable by the radar system [16,17,18].

Similarly, harmonic radar aids avalanche victim detection systems, identifying harmonic signals from reflectors often integrated into outdoor gear like ski clothing or backpacks [19]. Furthermore, it can be employed for cyclist detection by automotive radar [5].

Each application capitalizes on harmonic radar’s unique ability to detect specific, often small, objects in potentially complex settings. Tuning the system to transponders’ harmonics helps mitigate background noise, improving the signal-to-noise ratio and thus enhancing reliability and detection range.

This paper delves into practical implementation considerations and potential applications of the harmonic FMCW radar principle. This radar principle’s distinctive features render it particularly suitable for autonomous driving, surveillance systems, and aerospace applications.

## 3. Harmonic RFID Tags

**Figure 3:**
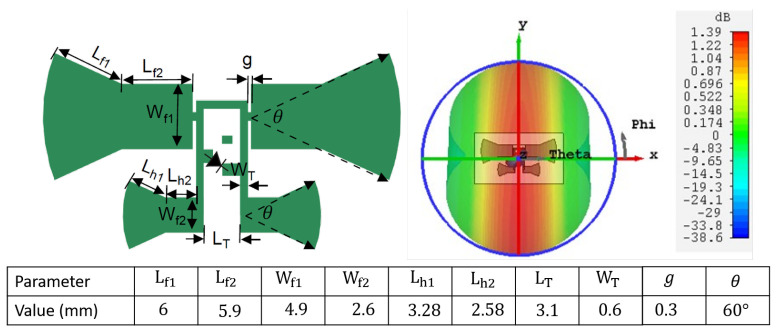
Harmonic transponder physical layout on the left, and the corresponding radiation pattern at 2.45 GHz on the right.

Chipless RFID tags grapple with clutter, which can be mitigated by introducing non-linearity into the tag’s antenna structure, generating higher harmonic frequencies. This differentiation allows the tag’s backscattered signal to be discerned from the environment. The existing literature indicates that incorporating a ground plane and matching it with lumped elements poses obstacles to tag printing, particularly when utilizing printable Schottky diodes [20]. Consequently, designing a single-layer, compact, lightweight, and efficient harmonic transponder becomes pivotal for full harmonic tag deployment. The utilized transponder comprises two bow tie dipoles matched to the diode via a T-matching network [21], as depicted in Figure 3. The planar T-matching network, unlike the distributed short-circuited stubs introduced in the literature [22], is compact and does not require grounded vias for diode matching, thus enjoying more acceptance.

**Figure 4:**
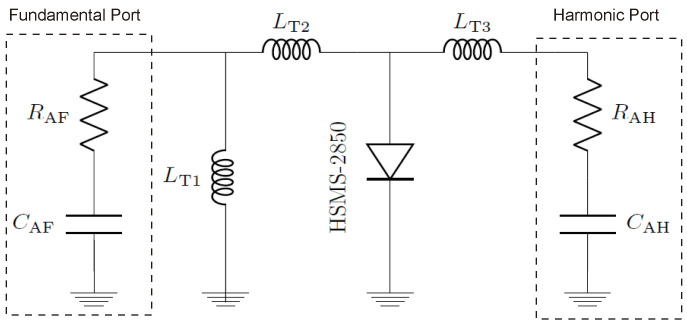
The simplified single-ended transponder circuit model.

**Figure 5:**
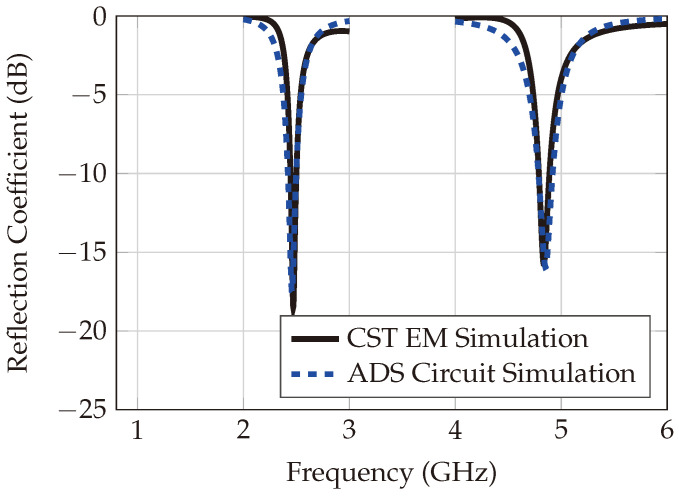
Circuit simulation vs. EM simulation.

The two dipoles serve as transponder antennas—one for receiving 2.45 GHz, and a smaller one for transmitting the converted first harmonic from the diode. The realized gains of the fundamental and harmonic antennas are 1.39 dBi and 3.8 dBi, respectively. The transponder circuit model is assessed for matching performance under various receiving powers and frequencies, as shown in Figure 4. Circuit and electromagnetic (EM) simulations align closely, as clarified in Figure 5.

## 4. System Model

**Figure 6:**
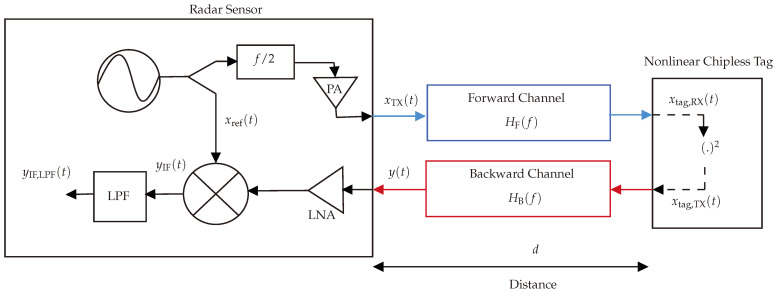
System model illustrating a graphical representation of the mathematical model.

The system model, as depicted in Figure 6, is mathematically defined as follows.

The transmitted FMCW radar signal is expressed as Equation (1) [23,24]:

(1) $$x_{\mathrm{TX}}\left(t\right)=A_{0}\cos\left[2\pi (f_{0}t+\mu t^{2})\right],$$

where $A_{0}$ denotes the signal amplitude, $f_{0}$ is the signal start frequency, and μ signifies the chirp slope, as expressed in Equation (2):

(2) $$\mu =\frac{B}{T_{\mathrm{sweep}}},$$

where *B* represents the signal bandwidth (chirp bandwidth), and $T_{\mathrm{sweep}}$ is the chirp sweeping time.

The forward and backward channel impulse responses characterize the radar channel’s responses in the forward and backward directions. In the context of multipath propagation, the transmitted signal reaches the harmonic tag as a combination of various delayed and attenuated versions, each following a distinct path. The forward and backward channel impulse responses are modeled using Equations (3) and (4), respectively. They are not identical due to the differing signal frequencies in the forward and backward channels:

(3) $$h_{F}\left(t\right)=\sum_{j=1}^{N^{F}}h_{j}^{F}\delta \left(t-t_{j}^{F}\right),$$

where $h_{j}^{F}$ denotes the path loss attenuation of the forward path *j*, $t_{j}^{F}$ is the time-of-flight for forward path *j*, and $N^{F}$ denotes the total number of forward paths; and

(4) $$h_{B}\left(t\right)=\sum_{i=1}^{N^{B}}h_{i}^{B}\delta \left(t-t_{i}^{B}\right),$$

where $h_{i}^{B}$ signifies the path loss attenuation of backward path *i*, $t_{i}^{B}$ is the time-of-flight for the backward path *i*, and $N^{B}$ denotes the total number of backward paths.

The signal received by the tag is influenced by the forward channel. Equation (5) incorporates the multipath effect:

(5) $$x_{\mathrm{tag},\mathrm{Rx}}\left(t\right)=\sum_{j=1}^{N^{F}}h_{j}^{F}A_{0}\cos\left[2\pi (f_{0}\left(t-\tau_{j}^{F}\right)+\mu {\left(t-\tau_{j}^{F}\right)}^{2})\right],$$

where $\tau_{j}^{F}$ represents the time-of-flight for the forward path *j*.

For simplicity, only the diode within the tag is considered, which squares the input signal to extract the harmonic terms, as shown in Equation (6).

(6) $$\begin{array}{ccc} {\left[x_{\mathrm{tag},\mathrm{Rx}}\left(t\right)\right]}^{2} & = & \sum_{j_{1}=1}^{N^{F}}h_{j_{1}}^{F}A_{0}\cos[2\pi (f_{0}\left(t-\tau_{j_{1}}^{F}\right)\\ & & \;\;\;\;\;+\mu {\left(t-\tau_{j_{1}}^{F}\right)}^{2})]\\ & & \times \sum_{j_{2}=1}^{N^{F}}h_{j_{2}}^{F}A_{0}\cos[2\pi (f_{0}\left(t-\tau_{j_{2}}^{F}\right)\\ & & \;\;\;\;\;+\mu {\left(t-\tau_{j_{2}}^{F}\right)}^{2})]\\ & = & \sum_{j_{1}=1}^{N^{F}}\sum_{j_{2}=1}^{N^{F}}h_{j_{1}}^{F}h_{j_{2}}^{F}A_{0}^{2}\cos[2\pi (f_{0}\left(t-\tau_{j_{1}}^{F}\right)\\ & & \;\;\;\;\;+\mu {\left(t-\tau_{j_{1}}^{F}\right)}^{2})]\\ & & \;\;\;\times \cos[2\pi (f_{0}\left(t-\tau_{j_{2}}^{F}\right)\\ & & \;\;\;\;\;+\mu {\left(t-\tau_{j_{2}}^{F}\right)}^{2})] \end{array}$$

Subsequently, the diode’s squaring is expressed in Equation (7), and the output signal from the diode is shown in Equation (8):

(7) $$\begin{array}{ccc} {\left[x_{\mathrm{tag},\mathrm{Rx}}\left(t\right)\right]}^{2} & = & \frac{A_{0}^{2}}{2}\sum_{j_{1}=1}^{N^{F}}\sum_{j_{2}=1}^{N^{F}}\{h_{j_{1}}^{F}h_{j_{2}}^{F}\cos[4\pi f_{0}t\\ & & \;\;\;\;\;-2\pi f_{0}\left(\tau_{j_{1}}^{F}+\tau_{j_{2}}^{F}\right)\\ & & \;\;\;\;\;+2\pi \mu {\left(t-\tau_{j_{1}}^{F}\right)}^{2}\\ & & \;\;\;\;\;+2\pi \mu {\left(t-\tau_{j_{2}}^{F}\right)}^{2}]\\ & & \;\;\;\;\;+\mathrm{DC}\} \end{array}$$

(8) $$\begin{array}{ccc} x_{\mathrm{tag},\mathrm{Tx}}\left(t\right) & = & \eta_{\mathrm{diode}}{\left[x_{\mathrm{tag},\mathrm{Rx}}\left(t\right)\right]}^{2}\\ & = & \frac{A_{0}^{2}}{2}\eta_{\mathrm{diode}}\sum_{j_{1}=1}^{N^{F}}\sum_{j_{2}=1}^{N^{F}}h_{j_{1}}^{F}h_{j_{2}}^{F}\cos[4\pi f_{0}t\\ & & \;\;\;\;\;-2\pi f_{0}\left(\tau_{j_{1}}^{F}+\tau_{j_{2}}^{F}\right)\\ & & \;\;\;\;\;+2\pi \mu {\left(t-\tau_{j_{1}}^{F}\right)}^{2}\\ & & \;\;\;\;\;+2\pi \mu {\left(t-\tau_{j_{2}}^{F}\right)}^{2}], \end{array}$$

where $\eta_{\mathrm{diode}}$ is the diode’s conversion loss, as discussed in [21,25]. Further details regarding antenna integration, polarization, and analog aspects are presented in [26]. The effect of the diode on the signal phase is not considered in the model or in this paper, since the utilized signal processing considers only the signal’s amplitude.

The signal received by the reader is influenced by the backward channel. It is important to note that the backward signal will experience greater attenuation (at least 6 dB more) due to the doubling of the signal frequency, resulting in increased path loss.

(9) $$\begin{array}{ccc} y(t) & = & y_{\mathrm{LOS}}\left(t\right)+y_{\mathrm{multipath}}\left(t\right)\\ & = & \sum_{i=1}^{N^{B}}h_{i}^{B}x_{\mathrm{tag},\mathrm{Tx}}\left(t-\tau_{i}^{B}\right)\\ & = & h_{1}^{B}x_{\mathrm{tag},\mathrm{Tx},\mathrm{LOS}}\left(t-\tau_{1}^{B}\right)\\ & + & \sum_{i=2}^{N^{B}}h_{i}^{B}x_{\mathrm{tag},\mathrm{Tx},\mathrm{multipath}}\left(t-\tau_{i}^{B}\right) \end{array}$$

(10) $$\begin{array}{ccc} y_{\mathrm{LOS}}\left(t\right) & = & h_{1}^{B}x_{\mathrm{tag},\mathrm{Tx},\mathrm{LOS}}\left(t-\tau_{1}^{B}\right)\\ & = & \frac{A_{0}^{2}}{2}\eta_{\mathrm{diode}}{\left(h_{1}^{F}\right)}^{2}h_{1}^{B}\cos[4\pi f_{0}\left(t-\tau_{1}^{B}\right)\\ & & -4\pi f_{0}\tau_{1}^{F}+4\pi \mu {\left(t-\tau_{1}^{B}-\tau_{1}^{F}\right)}^{2}]\\ & = & \frac{A_{0}^{2}}{2}\eta_{\mathrm{diode}}{\left(h_{1}^{F}\right)}^{2}h_{1}^{B}\cos[4\pi f_{0}(t-\\ & & \left(\tau_{1}^{F}+\tau_{1}^{B}\right))+4\pi \mu {\left(t-\left(\tau_{1}^{B}+\tau_{1}^{F}\right)\right)}^{2}] \end{array}$$

(11) $$\begin{array}{ccc} y_{\mathrm{multipath}}\left(t\right) & = & \sum_{i=2}^{N^{B}}h_{i}^{B}x_{\mathrm{tag},\mathrm{Tx},\mathrm{multipath}}\left(t-\tau_{i}^{B}\right) \end{array}$$

At the reader’s receiving antenna, the received signal primarily consists of two main components: the line-of-sight (LOS) segment and the multipath segment (which is reflected from surrounding objects).

The received signal, denoted as y(t), undergoes amplification by a low-noise amplifier and is then mixed with the reference signal $x_{\mathrm{ref}}\left(t\right)$ to yield an intermediate frequency (IF) signal, $y_{\mathrm{IF}}\left(t\right)$, as described in Equation (12):

(12) $$y_{\mathrm{IF}}\left(t\right)=y\left(t\right)x_{\mathrm{ref}}\left(t\right),$$

where

(13) $$x_{\mathrm{ref}}\left(t\right)=A_{\mathrm{ref}}\cos\left[2\pi (2f_{0}t+2\mu t^{2})\right],$$

and thus,

(14) $$y_{\mathrm{IF}}\left(t\right)=\sum_{k=1}^{K}A_{k}\cos\left(\frac{2B}{T_{\mathrm{sweep}}}\tau_{k}t+2\omega_{0}\tau_{k}-\frac{B}{T}\tau_{k}^{2}\right),$$

where $A_{k}$ represents the corresponding signal amplitude, encompassing the gain of the low-noise amplifier (LNA) as well as the conversion gain of the mixer. *B* signifies the FMCW sweeping bandwidth, *K* denotes the total reflected paths from the target (equal to $N^{F}+N^{B}$), $T_{\mathrm{sweep}}$ stands for the chirp sweeping time, and τ is the propagation delay defined as τ=(2.R/c), where *R* represents the range and *c* is the speed of light.

Subsequently, the signal is subjected to low-pass filtering to eliminate higher frequency components and to isolate the desired beat frequency. This process is detailed in Equation (15).

(15) $$y_{\mathrm{IF},\mathrm{LPF}}\left(t\right)=\sum_{k=1}^{K}A_{k}\cos\left(\frac{2B}{T_{\mathrm{sweep}}}\tau_{k}t-\frac{B}{T}\tau_{k}^{2}\right)$$

Consequently, the equation for range estimation can be expressed as follows:

(16) $$R=\frac{f_{\mathrm{IF},\mathrm{LPF}}T_{\mathrm{sweep}}c}{4B},$$

where $f_{\mathrm{IF},\mathrm{LPF}}$ represents the estimated beat frequency of the received signal after low-pass filtering. The frequency estimation of the signal is accomplished by employing a Fast Fourier Transform (FFT) operation.

Harmonic FMCW radar offers an advantage in range accuracy. Leveraging backscattered harmonic frequency ranges, this radar system achieves superior range accuracy relative to conventional FMCW radar. This improvement stems from the additional bandwidth that backscattered harmonic signals offer for enhanced target resolution. Therefore, the $B_{\mathrm{sweep}}$ value of 100 MHz is aptly employed for range determination, harmonizing well with measurements. The calculated distance *R* includes the forward and backward links. Therefore, the distance between the radar and the target is equal to R/2.

In terms of the link budget calculation, the second harmonic power received by the reader is given by Equation (17):

(17) $$P_{\mathrm{reader}-2\mathrm{ndH}}={\left(P_{t}\right)}^{2}{\left(G_{t}\right)}^{2}{\left(\frac{\lambda }{4\pi r}\right)}^{6}{\left(G_{\mathrm{tag}-\mathrm{rx}}\right)}^{2}\eta_{\mathrm{diode}}G_{\mathrm{tag}-\mathrm{tx}}G_{r},$$

where $P_{t}$ signifies the transmitted signal power, $G_{t}$ represents the transmitter antenna gain, *r* is the distance between the reader and the tag, λ is the wavelength, and $G_{\mathrm{tag}-\mathrm{rx}}$ represents the receiver antenna at the tag side.

## 5. Harmonic Radar Realization

**Figure 7:**
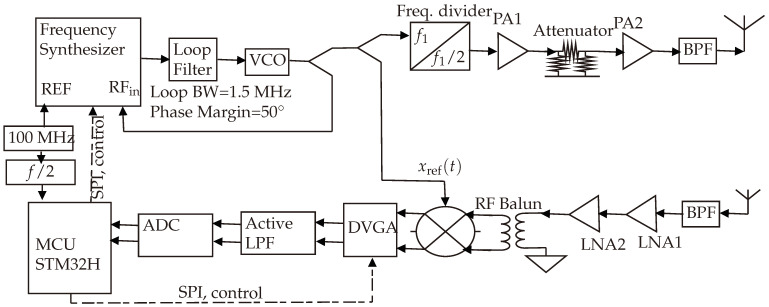
Detailed block diagram of the realized harmonic FMCW radar. The signal generated from the frequency synthesizer is an FMCW signal of frequency 4.8–5.0 GHz, which then passes through a frequency divider to generate the 2.4–2.5 GHz FMCW signal. This makes the reference signal as clean as possible.

Figure 7 provides an illustration of the implemented harmonic FMCW radar design, employed for detecting and estimating the range of the designed harmonic RFID tag.

### 5.1. Transmitter Chain

**Figure 8:**
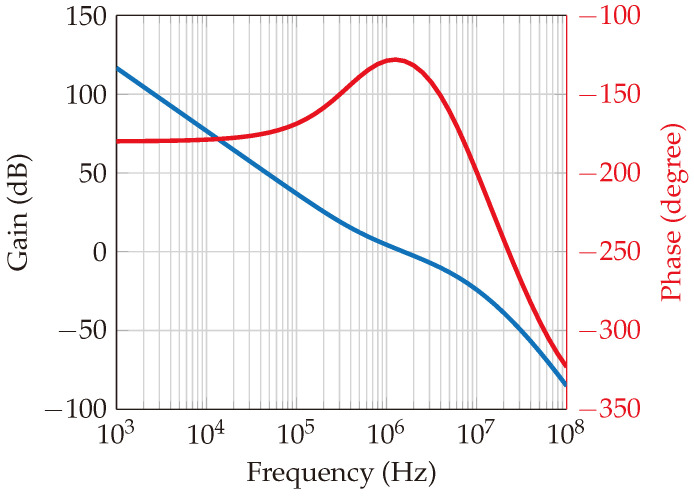
Loop filter open loop gain. The blue curve is the gain, and the red curve is the phase. The loop bandwidth happens in the crossover point of the gain curve, which is at 1.5 MHz. The phase margin is the value of phase at the crossover point (−180°); then, it will be around 50°, which indicates a stability in the PLL.

**Figure 9:**
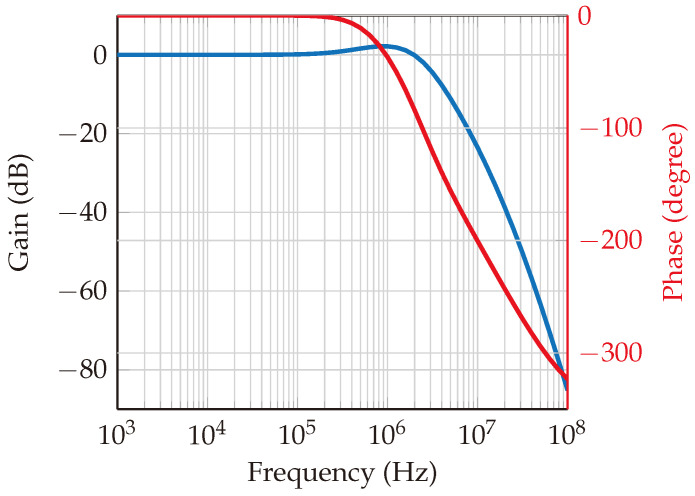
Loop filter closed loop gain. The blue curve is for gain (in dB), and the red curve is for phase (in degrees). The gain starts to decay at 1.5 MHz, and the corresponding phase margin is about 50°.

Within this system, a phase-locked loop (PLL) circuit is designed using the ADF4159 [27] frequency synthesizer chip with chirp/ramp functionality. The loop filter features a bandwidth of 1.5 MHz and a phase margin of 50°, as depicted in Figure 8 and Figure 9, which represent the open-loop and closed-loop gains, respectively. The loop bandwidth holds significance in a phase-locked loop (PLL) system, as it determines the range of frequencies over which the PLL can effectively track changes in the input signal. A wider loop bandwidth facilitates faster locking onto the input signal, albeit at the cost of increased susceptibility to noise. Conversely, a narrower loop bandwidth rejects more high-frequency noise while potentially prolonging the locking process. The choice of bandwidth necessitates a careful balance between noise performance and lock time. Additionally, the phase margin gauges the system’s resilience to variations in the phase of the input signal. A higher phase margin indicates superior stability, whereas a lower phase margin may result in instability. Further elaboration on the PLL’s functionality is provided in [28,29].

A wideband voltage-controlled oscillator (VCO)—HMC586 from Analog Devices [30]—was used to convert the sawtooth tuning voltage into an FMCW radar signal. As illustrated in Figure 7, the VCO is tuned to generate the harmonic frequency, which subsequently traverses an RF power splitter. One branch of the splitter feeds into the LO (local oscillator) terminal of the mixer, serving as the reference signal, denoted as $x_{\mathrm{ref}}$. The other branch of the splitter connects to an RF frequency divider to facilitate transmission of the fundamental frequency. Consequently, the transmitted signal spans from 2.4 to 2.5 GHz, resulting in a received signal with double the frequency range, i.e., from 4.8 to 5.0 GHz. This doubling of frequency range is attributed to the presence of a nonlinear component within the tag’s side, specifically the diode.

The generated wideband FMCW signal undergoes amplification and band-pass filtering, and it is then transmitted via the transmitting antenna. The attenuator, a digitally controlled, step-programmable attenuator, enables controlled variation of the transmitted power.

**Figure 10:**
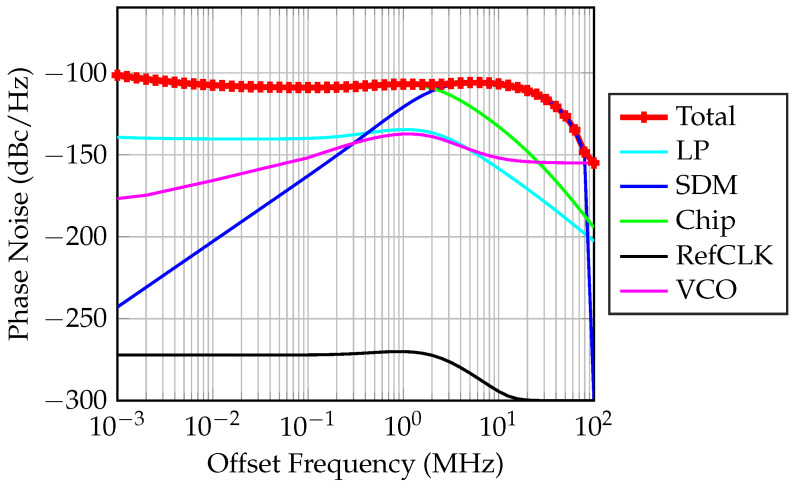
Total phase noise of the designed PLL. The intersection points of these noise contributions define the PLL’s phase noise floor and help in identifying the key areas for noise optimization to enhance the PLL’s performance in high-precision applications.

Phase noise within an FMCW radar system can significantly impact the accuracy of range measurements. This phenomenon affects the fidelity of both transmitted and received signals, leading to inaccuracies in estimating the range to a target. Phase noise manifests as deviations in the output frequency. Random fluctuations induced by phase noise in the instantaneous frequency of transmitted and received signals can deviate from the expected linear chirp waveform. Consequently, the measured beat frequency utilized for range determination may be affected, resulting in estimation errors and contributing to overall range inaccuracies. Extensive analysis of phase noise’s influence on FMCW systems is presented in [31,32,33,34]. The cumulative phase noise of the designed PLL is estimated and visualized in Figure 10. It encompasses phase noise from the LP (loop filter low-pass filter), the SDM (sigma-delta modulator), the chip (the ADF4159 chipset), Refclk (the reference clock used for the PLL), and the VCO. The total phase noise is quantified at −110 dBc/Hz.

### 5.2. Receiver Chain

The receiver chain plays a critical role in accurately extracting range information from received FMCW radar signals. Its meticulous design and optimization are crucial for achieving FMCW radar systems of high performance, boasting enhanced range accuracy, resolution, and target detection capabilities.

It is imperative to acknowledge that the precise implementation of the FMCW receiver chain could diverge, contingent on the system requirements, operational frequency, and the desired level of performance. Advanced FMCW radar systems may encompass additional components or employ specialized techniques to bolster sensitivity, nullify noise, or employ advanced signal processing.

The received signal initially undergoes bandpass filtering and subsequent amplification via a low-noise amplifier (LNA) featuring a noise figure of 1.5 dB. Following this, it is down-converted (dechirped) by employing the RF mixer, utilizing the reference signal $x_{\mathrm{ref}}$ as its local oscillator. The low noise figure of the LNA contributes to an overall reduction in the receiving chain’s total noise figure, which fundamentally influences receiver sensitivity.

To regulate the baseband signal’s amplitude, a differential variable gain amplifier (DVGA) is employed in tandem with an active low-pass filter. This combination serves to adapt the signal level to a magnitude suitable for the analog-to-digital converter (ADC). The total gain of the receiving channel is adjusted to surpass the ADC’s minimum detectable signal level (as illustrated in [35]) while remaining beneath the level that would lead to saturation [36].

Employed for data processing and transmission to the computer via USB, a robust m-7 microcontroller (STM32H7) demonstrates full synchronization with the PLL, as delineated in Figure 7.

**Figure 11:**
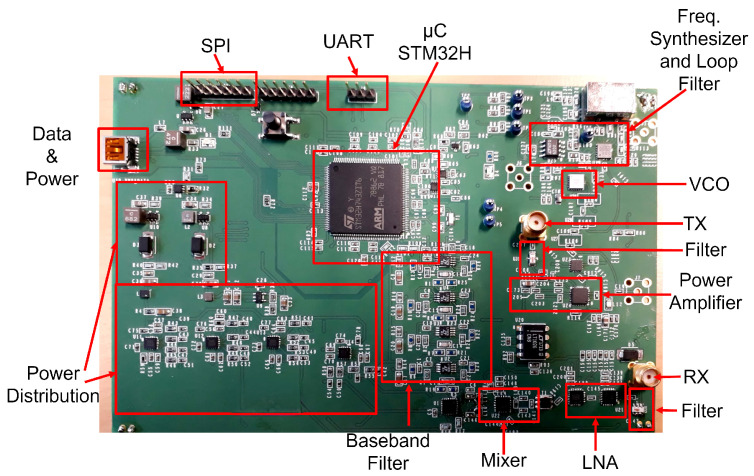
Manufactured harmonic FMCW radar. It is a 6-layer PCB architecture. The digital ground and the RF analog ground are separated and connected together in a single point to minimize the noise in both grounds.

Subsequently, the intricate block diagram is translated into circuits and printed circuit board layouts and eventually manufactured, as shown in Figure 11.

### 5.3. Power Consumption

**Figure 12:**
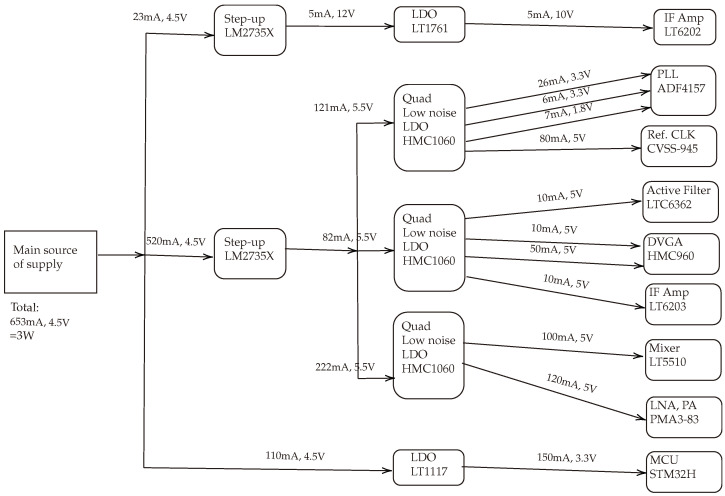
Power supply network illustration. In this chart, the main actual components are included to estimate the overall maximum power consumption.

Figure 12 shows a diagram of the power supply tree, detailing the distribution and individual power consumption of various components in the designed system. The main power supply delivers a total of 653 mA at 4.5 V, amounting to an overall power consumption of approximately 3 W, which could be easily delivered from a battery. This supply branches into several pathways, each with step-up converters or low-dropout regulators (LDOs) to adjust the voltage for specific components. This design guarantees a stable and low-noise DC voltages for all the critical RF and digital components.

The designed system could be powered up using batteries. To do so, the required battery capacity could be calculated. If we want the system to run for a certain number of hours *h*, we can calculate the required battery capacity in ampere-hours (Ah) using the formula: Battery Capacity(Ah)=Current(A)×Operating Time(h). Thus, for 4-h operation, the required battery capacity is calculated as 2.612 Ah. To have a margin, we might want to increase this capacity by at least 20–40% to account for battery aging, efficiency, and any additional consumption not accounted for in the initial design. Therefore, a battery with a capacity of around 3.5 to 4 Ah would be recommended.

## 6. Simulation Results

Within this section, the proposed mathematical framework introduced in Section 4 is subjected to simulation using the MATLAB R2022a tool, aiming to validate the utilized system. Figure 6 visually portrays the graphical representation of the simulated system model. The simulation incorporates a sawtooth ramp (chirp) with a sweeping time of 200 μs and a bandwidth of 200 MHz.

The backscattered signal is subsequently dechirped with a reference signal of twice the frequency before undergoing low-pass filtration. The signal then undergoes the Fast Fourier Transform (FFT) operation to isolate the beat frequency.

A straightforward peak detector algorithm is implemented, locating the initial peak of maximum magnitude and determining the corresponding frequency value. In MATLAB, the function *mspeaks* can serve this purpose. Subsequently, Equation (16) is utilized to convert the beat frequency into a range value.

**Figure 13:**
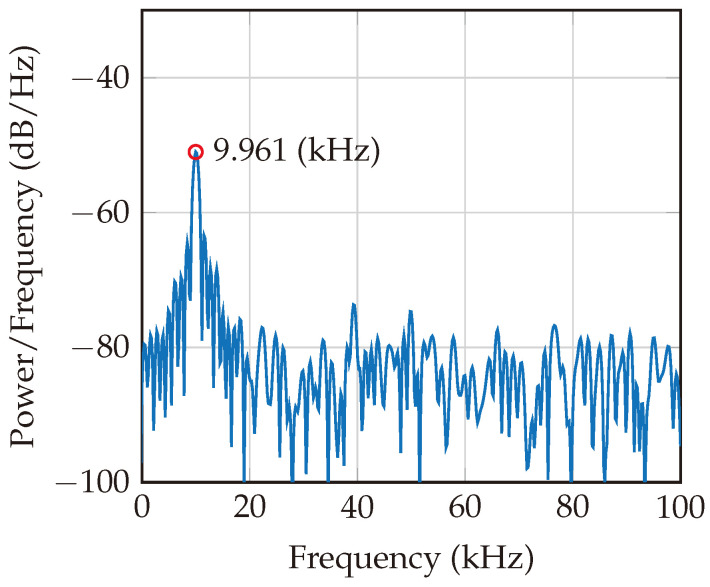
Beat frequency for a target at a distance of 0.37 m. The peak at 9.961 kHz indicates that there is one predominant harmonic tag existing at a distance of 0.37 m.

**Figure 14:**
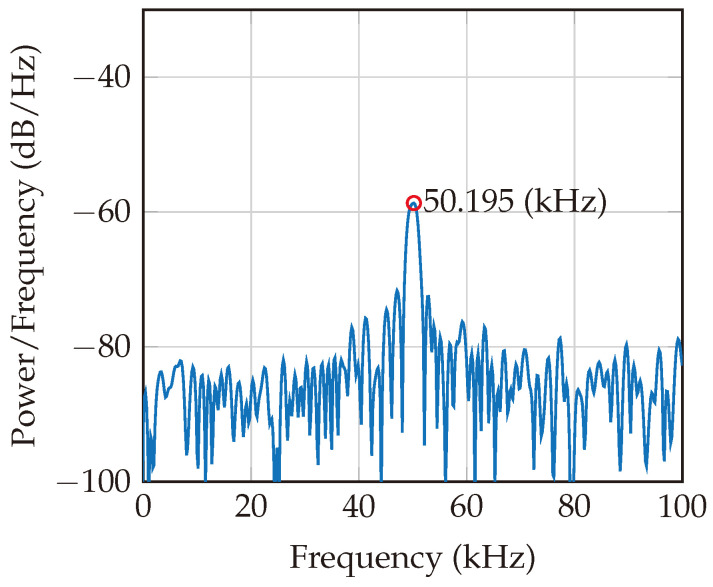
Beat frequency for a target at a distance of 1.87 m. The position of the peak is increased by increasing the harmonic tag distance.

The beat frequencies obtained in Figure 13 and Figure 14 are directly captured after the low-pass filtration for two different ranges (distances): 0.37 m and 1.87 m. These results consider the addition of AWGN to obtain an SNR of 10 dB.

While the MATLAB function *mspeaks* proves effective in scenarios featuring a clear peak within a minimally perturbed frequency spectrum (characterized by minimal multipath and noise), it is prudent to anticipate a more intricate peak detection algorithm to be introduced in a subsequent research paper.

It is important to note that the precise implementation of peak detection in FMCW radar systems is variable, contingent on system requisites and the radar signal’s characteristics. Advanced algorithms might incorporate sophisticated signal processing techniques, such as adaptive thresholding, multiple hypothesis testing, or machine learning methodologies, to enhance the accuracy and robustness of peak detection.

**Figure 15:**
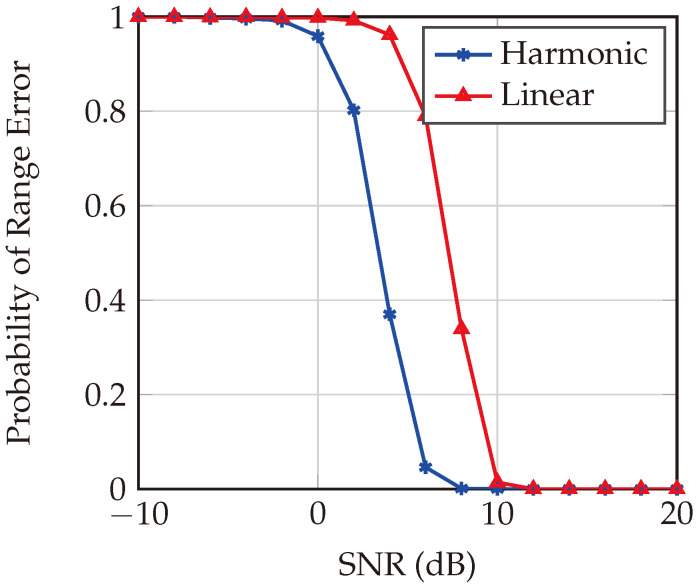
Probability of range error estimation for harmonic and linear FMCW systems in a multipath environment at a target distance of 1.87 m. The clutter-free environment and the wider bandwidth make the harmonic FMCW radar system outperform the traditional linear FMCW system.

The comparative performance of harmonic and linear FMCW radar systems within a multipath channel is meticulously evaluated, with Figure 15 illustrating the probability of range error across SNR values ranging from −10 dB to 20 dB for a target at a distance of 1.87 m. The figure reveals that, at low SNR levels (from −10 dB to approximately 0 dB), both systems exhibit high probabilities of range error, which is expected due to the increased noise relative to the signal that hampers precise range determination. Notably, between 0 dB and 10 dB SNR, the harmonic FMCW system demonstrates a gradual improvement, sustaining a lower probability of range error compared to its linear counterpart, suggesting superior noise resistance at these SNR levels. This enhanced performance can be attributed to two primary factors: the higher operational bandwidth of the harmonic system, which facilitates better range resolution, and its diminished susceptibility to clutter effects that significantly disrupt signal integrity in the linear FMCW system under low-SNR conditions. Ultimately, at SNR values exceeding 10 dB, both systems exhibit commendable performance.

## 7. Measurements

To validate the manufactured hardware and applied concepts, measurements were conducted on the VCO tuning voltage and the output signal’s spectrum. The tuning voltage, a pivotal parameter for VCO frequency control, must be within an appropriate range, with an accompanying control scheme to ensure the desired frequency’s accuracy, stability, and resolution for the specific application.

**Figure 16:**
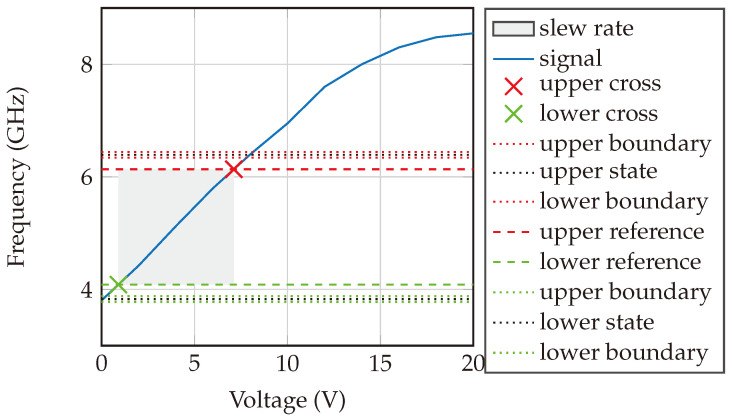
Slew rate of the VCO used to generate the sweeping signal. The slew rate is 329.93 MHz/V.

Figure 16 delineates the slew rate characteristics of the VCO implemented within our system. While the graph predominantly exhibits nonlinear frequency sweeping, a designated gray area distinctly demonstrates a near-linear response, aligning with our band of interest. This delineation is crucial, as it underpins our strategy for signal generation: the PLL is meticulously tuned to produce a frequency sweep ranging from 4.8–5 GHz. Subsequently, this signal is processed through a frequency divider, effectively yielding a lower frequency spectrum of 2.4–2.5 GHz. Notably, the slew rate of the VCO is precisely quantified at 329.93 MHz/V.

**Figure 17:**
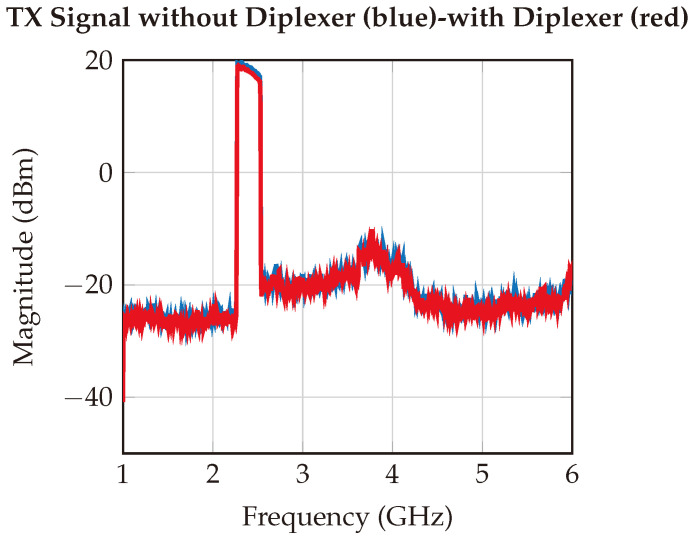
Measured output signal. The signal is measured by directly connecting an RF cable from the transmitter connector to the spectrum analyzer in order to investigate the power levels of the signal of interest and the harmonics.

Similarly, the output signal from the designed reader was measured using a spectrum analyzer. As demonstrated in Figure 17, the designed system effectively generates the required FMCW signal, maintaining a dynamic range of over 40 dB.

**Figure 18:**
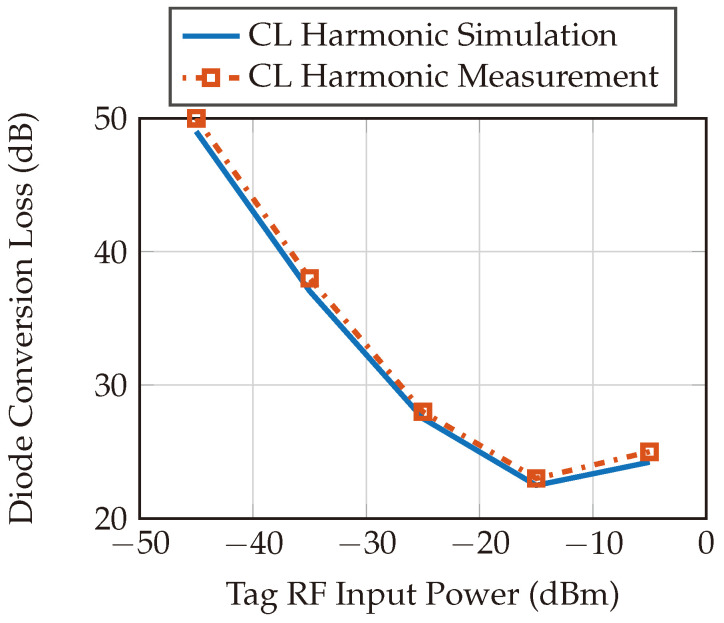
Diode conversion loss simulation and measurement results. The conversion loss is measured by designing a PCB that includes the diode. The diode’s input is matched to the fundamental frequencies, and the diode output is matched to the harmonic frequencies. A function generator is used to feed the diode input and vary the power, and a spectrum analyzer is used to capture the diode’s output power at the harmonic frequency.

Figure 18 shows the simulation and measurement results of the diode conversion loss integrated to the designed harmonic tag. The circuit illustrated in Figure 4 is simulated in PathWave’s Advanced Design System (ADS) simulation tool and then compared with the measured data.

The estimated SNR is 3.0 dB at the receiver’s antenna side, since the diode’s conversion loss will be −35 dBm, the designed receiver sensitivity is −121 dBm, the harmonic tag is at a distance of 1.7 m, and the transmitted signal power is 10 dBm EIRP. The SNR is calculated using Equation (18):

(18) $$\begin{array}{c} P_{\mathrm{sig}}=P_{t}-L_{2.4\mathrm{GHz}}+G_{\mathrm{tag},2.4\mathrm{GHz}}-\eta_{\mathrm{diode}}\\ +G_{\mathrm{tag},4.8\mathrm{GHz}}-L_{4.8\mathrm{GHz}}\\ P_{noise}=10\log\left(B_{\mathrm{baseband}}\right)-174-10\log\left(2\right)\\ \hat{\mathrm{SNR}}=P_{\mathrm{sig}}-P_{noise}, \end{array}$$

where $P_{\mathrm{sig}}$ is the signal power, $P_{t}$ is transmitted signal EIRP, $L_{2.4\mathrm{GHz}}$ is the free space path loss at the fundamental frequency, $G_{\mathrm{tag},2.4\mathrm{GHz}}$ is the tag’s receiving antenna gain, $\eta_{\mathrm{diode}}$ represents the diode’s conversion loss, $G_{\mathrm{tag},4.8\mathrm{GHz}}$ is the tag’s transmitting antenna gain, $L_{4.8\mathrm{GHz}}$ is the free space path loss at the harmonic frequency, $P_{noise}$ is the estimated noise power, $B_{\mathrm{baseband}}$ is the bandwidth of the baseband low-pass filter, and $\hat{\mathrm{SNR}}$ is the estimated SNR. It is worth noting that all the designed signal paths are differential, resulting in an enhancement in the noise performance by 10log(2) dB.

### 7.1. Measurement Setup

**Figure 19:**
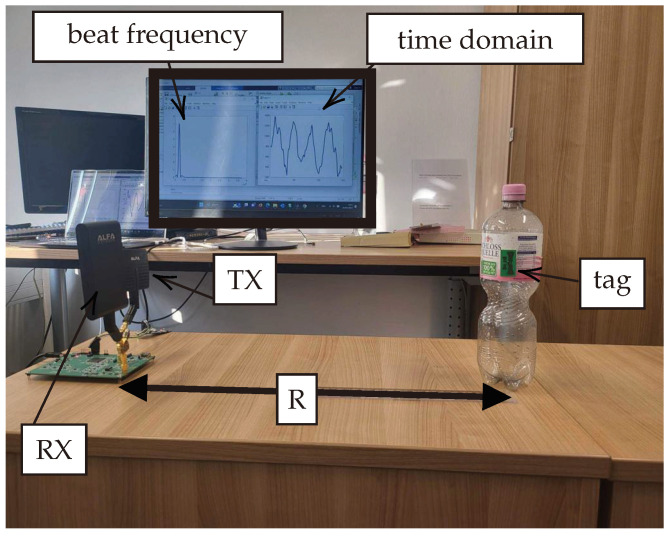
Measurement setup. The designed system is connected to a laptop to share the data and plot them. The real-time data that appear on the screen represent the time domain signal and the corresponding frequency domain beat signal that corresponds to the measured distance.

The measurement setup illustrated in Figure 19 was deployed to capture the measured signals. On the transmitter side, an antenna with 7 dBi gain (APA-M04 [37]) operates within the fundamental frequency range (2.4 to 2.5 GHz). Conversely, an 8 dBi antenna (APA-M25-6E [38]) is employed on the receiver side within the harmonic frequency range (4.8 to 5.0 GHz). The transmitted signal power is 10 dBm EIRP.

The designed harmonic radar board draws power from the USB, with raw data sent from the USB in real-time, as illustrated in Figure 11. These data are transmitted to a laptop for FFT operations on each up and down ramp.

For optimal ADC performance, the internal ADC of the utilized microcontroller is employed, boasting 16-bit resolution and a sampling rate of 744 ksps. A direct memory access (DMA) interface enhances data exchange, with processed data interfaced and processed in real-time.

Transmitted power is calibrated to an effective isotropic radiated power (EIRP) of 10 dBm to meet regulatory requirements for short-range devices (SRD). The ramp time is set to 200 μs, and the transmitted FMCW signal maintains a bandwidth of 100 MHz throughout range estimation measurements.

The passive tag is mounted on an empty bottle of water to evaluate the impact of positioning the tag on plastic material, deliberately excluding the water’s dielectric properties from our initial considerations. Potential clutter interference is hypothesized to originate from nearby metallic objects or the concrete structure of the surrounding environment. Looking ahead, we plan to extend our investigation by mounting the passive tag on a variety of real-world products, each characterized by distinct dielectric properties. This future work aims to assess the system’s performance across a broader spectrum of conditions and define the operational limits accordingly. To ensure the utmost accuracy in our measurements, the position of the target (the passive tag mounted on the water bottle) was precisely determined using a Bosch GLM 500 laser meter. This instrument enabled us to ascertain the exact distance between the tag and the system’s TX/RX antennas, ensuring that our data reflect true distances with high fidelity.

The number of measurements performed per tag position is 500 times. This is to estimate the range error accurately and to test the robustness of the system.

### 7.2. Measurement Results

The beat frequency serves as the foundation for nonlinear (harmonic) target range calculations. Analyzing the beat frequency over time permits the radar system to measure the time delay between transmitted and received signals, thereby furnishing insights into target distance.

The beat frequency and range/distance are inextricably linked. The connection is mathematically expressed in Equation (16).

**Figure 20:**
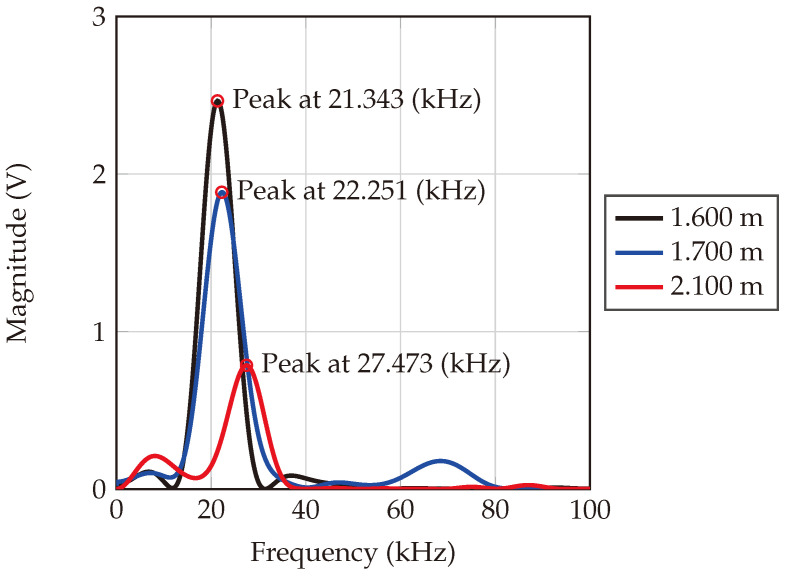
Frequency domain of the measured beat frequency. The peak of the black curve is at a frequency of 21.343 kHz, which shows a distance of 1.599 m. The blue curve is at a frequency of 22.251 kHz, which indicates a distance of 1.669 m. The red curve is at a frequency of 27.473 kHz, which indicates a distance of 2.059 m.

Measurements were conducted at three different distances: 1.600 m, 1.700 m, and 2.100 m. The corresponding beat frequencies are 21.343 kHz, 22.251 kHz, and 27.343 kHz, respectively, as depicted in Figure 20. Each peak in the data corresponds to a beat frequency that uniquely represents a specific distance between the radar and its target. Notably, as the distance measured increases, so does the frequency position of each peak. Furthermore, the observed decay in signal magnitude between the distances of 1.600 m and 1.700 m is minimal, attributable to the mere 10 cm difference between them. Conversely, a significantly greater magnitude decay is observed at a distance of 2.100 m, reflecting the larger distance from the radar.

**Figure 21:**
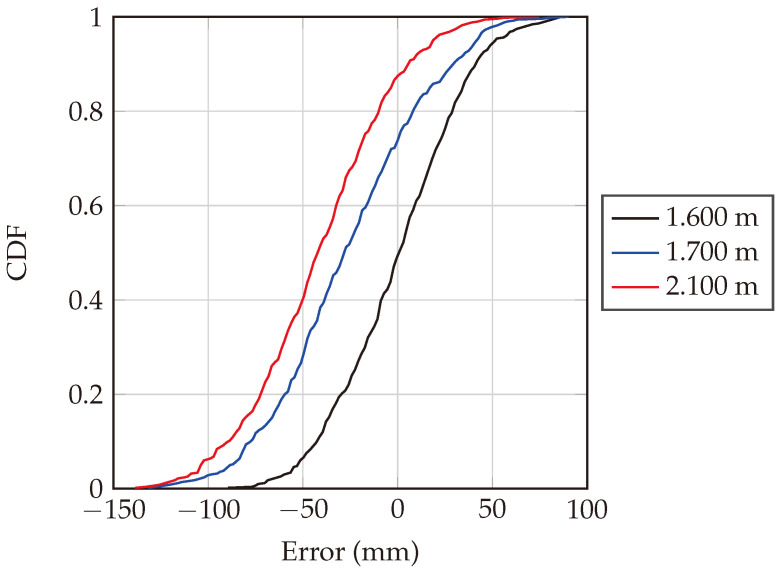
Cumulative distribution function of the ranging error at actual distances of 1.600 m, 1.700 m, and 2.100 m. The number of measurements made to plot this graph is 500, which also validates the system robustness.

The cumulative distribution function (CDF) is calculated for the 500 measurement iterations for the aforementioned tag positions, as depicted in Figure 21. The CDF function shows the probability that a variable is less than or equal to a certain value. For a given value on the x-axis (error in mm), the CDF value on the y-axis indicates the fraction of measurements that were below that error threshold. As observed from Figure 21, the black curve (1.600 m dataset) is closest to the center, indicating that measurements at this distance had a lower error compared to the other two distances. Moreover, the median error, which corresponds to CDF equal to 0.5, is 3.92 mm. Applying the same for the other datasets, then, the 1.700 m dataset provides a larger error, with a median error of −26.1 mm and finally 2.100 m dataset offers the largest error with a median error of −42.8 mm. Table 1 succinctly summarizes the median of the absolute errors of the measured distances for each actual distance for the 500 measurements, with actual distances gauged using a Bosch GLM 500 laser meter measuring device.

To further validate the proposed system, measurements have been expanded to include additional distance values, as detailed in Table 2. The comparison presented in the table demonstrates a close alignment between the measured distances and their actual counterparts, affirming the system’s accuracy.

Table 3 offers a comprehensive comparison of various harmonic radar systems, including the proposed system. It provides a detailed overview of technological specifications, such as waveform types, transmission and reception frequencies, signal bandwidth, antenna gains, and operational ranges. As depicted in the table, the proposed system is tailored for short-range applications with a passive design, making it potentially useful for scenarios where low power consumption and short ranges are desired or sufficient.

## 8. Conclusions

This paper presents the development of a wideband harmonic frequency-modulated continuous wave (FMCW) radar system, constructed using off-the-shelf components. The system is strategically designed to utilize the free industrial, scientific, and medical (ISM) frequency band (2.4–2.5 GHz) for the forward channel and the ultra-wide band (UWB) frequency band (4.8–5.0 GHz) for the backward channel. In addition, we have developed a wideband passive harmonic tag, designed for attachment to objects, facilitating their detection by the harmonic FMCW radar. A comprehensive mathematical framework is also established to characterize the harmonic radar system’s performance in line-of-sight (LOS) and multipath conditions, enabling an evaluation of its efficacy in overcoming clutter and multipath reflections. Validation of the system was conducted through measurements aimed at estimating the distances of objects, showcasing the system’s ability to provide precise range estimations in indoor settings. Owing to its low power consumption, which is attributed to minimal power requirements for transmission, the system is deemed highly suitable for a broad spectrum of short-range applications.

## Figures and Tables

**Table 1:** Range accuracy for 500 measurements.

Actual Distance (m)	Absolute Median Error (mm)
1.600	3.92
1.700	26.1
2.100	42.8

**Table 2:** Measured distance at several ranges with the corresponding measured beat frequencies.

Actual Distance (m)	Measured Beat Frequency (kHz)	Measured Distance (m)
1.000	13.663	1.024
1.200	15.746	1.181
1.600	21.343	1.669
1.800	24.126	1.811
2.000	25.898	1.942
2.100	27.473	2.059

**Table 3:** Comparison between state-of-the-art harmonic radar systems and the proposed one. This table is adapted from [1].

Ref., Year	Waveform Type	TX/RX Freq. (GHz)	$\mathrm{BW}{\;}^{\$}$ (GHz)	$P_{\mathrm{TX}}$ (dBm)	Reader		Tag			Range (m)
					$G_{\mathrm{TX}}^{f}$ (dBi)	$G_{\mathrm{RX}}^{h}$ (dBi)	$G_{\mathrm{TX}}^{f}$ (dBi)	$G_{\mathrm{RX}}^{h}$ (dBi)	Type	
**[This]**	**FMCW**	**2.4/4.8**	**0.2**	**3**	**7**	**8**	**1.39**	**3.8**	**passive**	**3**
[7], 2020	FMCW	2.9/5.8	0.16	34.7	13	14	-	-	passive	40
[5], 2023	FMCW	79/158	4	9	28	34	12	12	active	80
[1], 2021	FMCW	61/122	8	6	21	22.5	7.5	7.1	active	23
[7], 2020	FMCW	9.3/18.6	0.16	40	15	15	-	-	passive	15
[8], 2008	FMCW	5.95/11.9	0.2	20	22	22	5.5	2.8	passive	58
[6], 2013	CW	38.5/77	-	21	25	24	6.73	6.71	active	1
[39], 2016	$\mathrm{PRN}{\;}^{\&}$	9.4/18.8	0.025	32.4	38	43	0.2	0.2	passive	180

$ Bandwidth of the backscattered harmonic signal; & Pseudorandom noise code.

## Data Availability

Data are contained within the article.
